# Tracking control problem of nonlinear strict-feedback systems with input nonlinearity: An adaptive neural network dynamic surface control method

**DOI:** 10.1371/journal.pone.0312345

**Published:** 2024-10-24

**Authors:** Minglong Zhou, Xiyu Zhang, Xiongfeng Deng

**Affiliations:** 1 School of Electrical Engineering, Anhui Technical College of Mechanical and Electrical Engineering, Wuhu, China; 2 Zhejiang Dongfang Polytechnic, Wenzhou, China; 3 Key Laboratory of Electric Drive and Control of Anhui Higher Education Institutes, Anhui Polytechnic University, Wuhu, China; University of Shanghai for Science and Technology, CHINA

## Abstract

In this work, the tracking control problem for a class of nonlinear strict-feedback systems with input nonlinearity is addressed. In response to the influence of input nonlinearity, an auxiliary control system is constructed to compensate for it. To process unknown nonlinear dynamics, radial basis function neural networks (RBFNNs) are introduced to approximate them, and some adaptive updating control laws are designed to estimate unknown parameters. Furthermore, during the dynamic surface control (DSC) design process, first-order low-pass filters are introduced to solve the complexity explosion problems caused by repeated differentiation. After that, an NN-based adaptive dynamic surface tracking controller is proposed to achieve the tracking control. By applying the proposed controller, it can be guaranteed that not only the output of the system can track the desired trajectory, but also that the tracking error can converge to a small neighborhood of zero, while all signals of the closed-loop system are bounded. Finally, the effectiveness of the proposed controller is verified through two examples.

## 1. Introduction

In the past decades, a large number of control methods have emerged, such as sliding mode control method [1], PID method [2], adaptive control method [3], fuzzy control method [4,5], and NN control method [6], among which the NN control method has received widespread attention. Due to the effective approximation of unknown nonlinear dynamics, the NN control method has been used to solve the control problems of various uncertain nonlinear systems. For example, the authors of [7] solved the approximation problem of uncertain nonlinear dynamics caused by the deviation of control parameters from the actual values. In [8], an adaptive NN tracking control strategy with backstepping control technique was designed to achieve the tracking control. Furthermore, the authors of [9] proposed an NN-based exponential feedback control method, in which the output feedback control issue of a calss of strict-feedback nonlinear systems with different time-delay states was solved. Moreover, the fuzzy-neural control method addressed in [10] solved the prescribed performance control problem of constrained wave-rider vehicles.

On the other hand, as an important part of a control system, the occurrence of relevant faults in actuators will impact the system control effect and may even lead to the system instability when serious. Among actuator faults, the input saturation fault has always been a hot topic for scholars. Designing effective control schemes for nonlinear systems with input saturation faults is a key research direction. In [11], the authors considered the fault-tolerant control problem of a class of Takagi-Sugeno fuzzy system with actuator faults and saturation, where an adaptive observer-based fault-tolerant control strategy was addressed. In [12], the authors developed an adaptive DSC strategy with fuzzy-neural control method, where the tracking error can converge in a finite time under the designed control strategy. In [13], the authors proposed a smooth non-affine function that can control the input signal to solve the approximation problem of the input saturation function in the given system. In [14, the authors addressed the trajectory tracking problem of a class of autonomous underwater vehicles with model uncertainties and input saturation, in which the proposed adaptive command filtered backstepping control strategy can ensure the boundedness of all signals. Furthermore, an improved event-triggered command filter backstepping tracking control scheme were further presented by the authors of [15], in which the event-triggered tracking problem of uncertain nonlinear systems under input saturation was solved. Additionally, for nonlinear systems with unknown dead-zone input [16,17], unknown input saturation [18], and unknown control gains [19–21] are also of great interest of researchers. However, it should be noted that in practical control systems, the limited amplitude of actuator output means that actuator saturation can affect the stability and dynamic performance. Therefore, while researching the tracking control problem of nonlinear systems, considering the existence of input saturation faults is of great practical significance.

As discussed in [22–24], the backstepping control method has been widely used to tackle various control issues of complex nonlinear systems. However, this method requires repeated differentiation of the designed virtual control law, which may lead to the “complexity explosion” problem. In order to solve this drawback, many scholars have explored research from different perspectives. In [25,26], the authors introduced a DSC method to replace the traditional backstepping control method, and proposed an adaptive DSC strategy to solve the control problem of nonlinear systems with sensor faults. In [27], the authors designed an adaptive backstepping DSC scheme that effectively eliminated the “complexity explosion” problem caused by repeated derivation of the virtual control law. It is worth noting that the authors of [27] introduced an unknown function in the design of the controller, which may lead to the occurrence of oscillation phenomena. In [28], although the proposed controller performed well for the given system, the introduction of the sign function in the controller design may result in control signal divergence. To this end, in [29], a class of sliding mode filter was introduced to improve the DSC method, where the authors proposed an adaptive fuzzy DSC method and solved the adaptive tracking control problem of a class of uncertain non-affine nonlinear systems. Meanwhile, the authors of [30] further combined the DSC method with nonlinear filters, and proposed a filter-based adaptive DSC strategy. Moreover, the authors of [31] introduced the nonlinear function approximation technique into the DSC design, and improved the control effect while simplifying the controller design process. Similarly, the authors of [32,33] addressed uncertain nonlinear dynamics of given systems from the perspectives of fuzzy logic system and NN, where the proposed tracking control methods not only achieved excellent tracking performance but also ensured strong robustness.

In light of the above analysis, it is not difficult to find that there are not many discussions on the nonlinear strict-feedback systems with input saturation. Therefore, this work discusses the tracking control problem of a type of strict-feedback nonlinear system with input saturation nonlinearity. The impact of input saturation is handled by constructing a type of auxiliary dynamic systems. By introducing the DSC method and RBFNN approximation technique, the parameter adaptive updating control laws and an NN-based adaptive DSC controller are proposed. Overall, the main contributions of this work include:

- An auxiliary dynamic system is constructed to compensate for the input saturation nonlinearity of the studied strict-feedback nonlinear systems. Compared with [13–15], the introduction of auxiliary control systems not only reduces the difficulty of handling input saturation nonlinearity, but also reduces the difficulty of controller design.
- Different from [25–27], this work simultaneously considering dynamic surface control method and neural network approximation technique, the problems caused by repeated differentiation and unknown nonlinear dynamics are effectively solved. Furthermore, it effectively overcomes the issue of computational complexity.
- An NN-based adaptive dynamic surface tracking control strategy is proposed for the tracking problem. It can be ensured that the system tracking error can converge to a small neighborhood of zero, and all signals of the closed-loop system are bounded.

The following arrangements of this work are as follows. In Section 2, the problem description and some useful assumptions and lemmas are provided. In Section 3, as the important part of this work, involves the design and stability analysis of the adaptive tracking controller. Section 4 presents two simulation examples to illustrate the theoretical analysis results; and finally, brief conclusions of this work are presented in Section 5.

## 2. Problem description and preliminaries

### Problem description

Consider a class of strict-feedback nonlinear systems with input saturation nonlinearity, which is described as

$$\left\{ \begin{array}{l} {\dot{x}}_{i}=g_{i}({\bar{x}}_{i})x_{i+1}+f_{i}({\bar{x}}_{i})+d_{i}(t),1\leq i\leq n-1\\ {\dot{x}}_{n}=g_{n}({\bar{x}}_{n})Q(u(t))+f_{n}({\bar{x}}_{n})+d_{n}(t)\\ y=x_{1} \end{array}\right.$$ (1)

where ${\bar{x}}_{i}={\left[x_{1},\cdots ,x_{i}\right]}^{T}\in R^{i}$ (*i* = 1,⋯,*n*) and *y*∈*R* represent the state vector and output of the system, *Q*(*u*(*t*)) is the control input, $f_{i}({\bar{x}}_{i})$ (*i* = 1,⋯,*n*) represent the unknown nonlinear functions, $g_{i}({\bar{x}}_{i})\neq 0$ (*i* = 1,⋯,*n*) are the control gains and represent known time-varying continuous functions, and *d*_*i*_(*t*)(*i* = 1,⋯,*n*) represent unknown external disturbances. For the convenience of subsequent analysis, without causing confusion, this work abbreviates $f_{i}({\bar{x}}_{i})$, $g_{i}({\bar{x}}_{i})$ and *d*_*i*_(*t*) as *f*_*i*_, *g*_*i*_ and *d*_*i*_, respectively.

In this work, it is assumed that the system control input *Q*(*u*(*t*) is subjected to actuator saturation fault [15,34], which is described as

$$Q(u(t))=\{\begin{array}{cc} U_{\max} & u(t)\geq U_{\max}\\ u(t) & U_{\min}<u(t)<U_{\max}\\ U_{\min} & u(t)\leq U_{\min} \end{array}$$ (2)

where *U*_max_ and *U*_min_ represent the upper and lower bounds of input saturation faults, respectively.

The main control objective of this work is to design an adaptive NN dynamic surface tracking controller for the system (1), such that the system’s output can track the given reference trajectory, and the tracking error converges to a small neighborhood of zero, while ensuring that all signals of the closed-loop system are bounded.

Assumption 1

The external disturbances *d*_*i*_ (*i* = 1,⋯,*n*) are bounded, that is, there exist positive constants *d*_*iM*_ and satisfy |*d*_*i*_|≤*d*_*iM*_.

Assumption 2

The given reference trajectory *y*_*d*_ and its the *i*th derivative exists and is smooth, bounded, and there exists a positive constant Ξ_1_, such that $\Omega_{1}≔\{(y_{d},{\dot{y}}_{d},\cdots ,y_{d}^{\left(i\right)}):y_{d}^{2}+{\dot{y}}_{d}^{2}+\cdots +{\left(y_{d}^{\left(i\right)}\right)}^{2}\leq \Xi_{1},i=1,\cdots ,n\}$.

Assumption 3

The control gains *g*_*i*_ (*i* = 1,⋯,*n*) are bounded, and there exists a positive constant *g*_0_, such that 0<|*g*_*i*_|≤*g*_0_. Without loss of generality, we assume *g*_*i*_>0.

### RBFNN

To deal with the unknown nonlinear dynamics generated in control design, an RBFNN is introduced for approximation processing. Considering a continuous unknown nonlinear function *h*(*Z*), and applying an RBFNN, then we have

$$h(Z)=W^{T}S(Z)$$ (3)

where *Z*∈Ω_*Z*_⊂*R*^*n*^ is the input vector of the NN, *W* = [*w*_1_,⋯,*w*_*l*_]^*T*^∈*R*^*l*^ represents the weight vector, and *l*>1 is the number of nodes, *S*(*Z*) = [*s*_1_(*Z*),⋯,*s*_*l*_(*Z*)]^*T*^∈*R*^*l*^ represents the radial basis function vector, and *s*_*i*_(*Z*)(*i* = 1,⋯,*l*) is usually chosen as a Gaussian function, that is

$$s_{i}(Z)=\exp(-\frac{{\left(Z-\chi_{i}\right)}^{T}(Z-\chi_{i})}{b_{i}^{2}}),i=1,\cdots ,l$$ (4)

where *χ*_*i*_ = [*χ*_*i*1_,⋯,*χ*_*in*_]^*T*^∈*R*^*n*^ and *b*_*i*_ represent the center and the width of radial basis function, respectively.

If the ideal weight vector W* is selected to have the minimum approximation error [8,33], then we have

$$W^{*}=\arg\underset{W\in R^{l}}{\min}\{\underset{Z\in \Omega_{Z}}{\sup}\left|h(Z)-W^{T}S(Z)\right|\}$$ (5)

and

$$h(Z)={\left(W^{*}\right)}^{T}S(Z)+\varepsilon (Z)$$ (6)

where *ε*(*Z*) is approximation error and satisfies $|\varepsilon (Z)|\leq \bar{\varepsilon }$ with $\bar{\varepsilon }$ being a positive constant.

**Lemma 1** [35]. For any *X*∈*R*^*n*^, *Y*∈*R*^*n*^, *p*>0 and *q*>0, if satisfies (*p*−1)(*q*−1) = 0, then the following inequality holds

$$XY\leq \frac{1}{p}{\left|X\right|}^{p}+\frac{1}{q}{\left|Y\right|}^{q}$$ (7)

**Lemma 2** [36. For any *b*∈*R* and *η*>0, the hyperbolic tangent function tanh(∙) satisfies

$$0\leq |b|-b\tanh(\frac{b}{\eta })\leq 0.2785\eta$$ (8)

## 3. Controller design and stability analysis

### Tracking controller design

In order to achieve the tracking control problem of the strict-feedback nonlinear systems with input saturation nonlinearity, this section combines DSC method and NN control method to design relevant parameter adaptive updating control laws and tracking controller. In the first *n*−1 step, RBFNNs are used to approximate the unknown nonlinear dynamics, and corresponding parameter updating control laws are designed for the unknown parameters. In the final step, the adaptive NN dynamic surface tracking controller is proposed. For the convenience of analysis in the following sections, the time variable *t* will be ignored.

To handle input saturation nonlinearity in the system (1), the following auxiliary dynamic system is designed.

$$\left\{ \begin{array}{l} {\dot{\lambda }}_{i}=-c_{i}\lambda_{i}+g_{i}\lambda_{i+1},i=1,\cdots ,n-1\\ {\dot{\lambda }}_{n}=-c_{n}\lambda_{n}+g_{n}\Delta u\\ \Delta u=Q(u(t))-u(t) \end{array}\right.$$ (9)

where *λ*_*i*_(*i* = 1,⋯,*n*) are the states of the auxiliary system, *c*_*i*_>0(*i* = 1,⋯,*n*) are design parameters.

Hence, we can define the following error coordinate transformation, that is

$$\left\{ \begin{array}{l} z_{1}=x_{1}-y_{d}-\lambda_{1}\\ z_{i}=x_{i}-{\bar{\alpha }}_{i-1}-\lambda_{i},i=2,\cdots ,n \end{array}\right.$$ (10)

where *z*_*i*_(*i* = 1,⋯,*n*) represent the error surfaces, ${\bar{\alpha }}_{i-1}$ (*i* = 2,⋯,*n*) are filter’s output with input being virtual control laws *α*_*i*−1_ which will be provided in subsequent analysis, *λ*_*i*_(*i* = 1,⋯,*n*) are the auxiliary states defined in (9), and *y*_*d*_ is the given reference trajectory.

#### Step 1

According to (1), ${\dot{\lambda }}_{1}=-c_{1}\lambda_{1}+g_{1}\lambda_{2}$ and (10), the time derivative of *z*_1_ can be expressed as

$${\dot{z}}_{1}=g_{1}z_{2}+g_{1}{\bar{\alpha }}_{1}+f_{1}+d_{1}-{\dot{y}}_{d}+c_{1}\lambda_{1}$$ (11)

Noting *f*_1_ is an unknown nonlinear function, and an RBFNN is introduced to approximate it, then we get

$$f_{1}={\left(W_{1}^{*}\right)}^{T}S_{1}(Z_{1})+\varepsilon_{1}(Z_{1})$$ (12)

where *ε*_1_(*Z*_1_) is approximation error and satisfies $|\varepsilon_{1}(Z_{1})|\leq {\bar{\varepsilon }}_{1}$ with ${\bar{\varepsilon }}_{1}>0$.

Substituting (12) into (11) gets

$${\dot{z}}_{1}=g_{1}z_{2}+g_{1}{\bar{\alpha }}_{1}+{\left(W_{1}^{*}\right)}^{T}S_{1}(Z_{1})-{\dot{y}}_{d}+c_{1}\lambda_{1}+\varepsilon_{1}(Z_{1})+d_{1}$$ (13)

Due to $|\varepsilon_{1}(Z_{1})|\leq {\bar{\varepsilon }}_{1}$ and |*d*_1_|≤*d*_1*M*_, then $|\varepsilon_{1}(Z_{1})+d_{1}|\leq {\bar{\varepsilon }}_{1}+d_{1M}$ and let $D_{1}={\bar{\varepsilon }}_{1}+d_{1M}$, where *D*_1_ is an unknown positive constant.

To avoid the problem of complexity explosion caused by repeated differentiation, a first-order low-pass filter 1/(*τ*_1s_+1) with time constant *τ*_1_ being introduced, then we obtain

$$\tau_{1}{\dot{\bar{\alpha }}}_{1}+{\bar{\alpha }}_{1}=\alpha_{1},\alpha_{1}(0)={\bar{\alpha }}_{1}(0)$$ (14)

where the virtual control law *α*_1_ as the input and the newly introduced state ${\bar{\alpha }}_{1}$ as the output.

Defining the filtering error *β*_1_ as

$$\beta_{1}={\bar{\alpha }}_{1}-\alpha_{1}$$ (15)

Combining (14) and (15), we have

$${\dot{\bar{\alpha }}}_{1}=\frac{\alpha_{1}-{\bar{\alpha }}_{1}}{\tau_{1}}=-\frac{\beta_{1}}{\tau_{1}}$$ (16)

Considering ${\bar{\varepsilon }}_{1}+d_{1M}=D_{1}$, and substituting (15) into (13) yields

$${\dot{z}}_{1}\leq g_{1}z_{2}+g_{1}\alpha_{1}+{\left(W_{1}^{*}\right)}^{T}S_{1}(Z_{1})+g_{1}\beta_{1}-{\dot{y}}_{d}+c_{1}\lambda_{1}+D_{1}$$ (17)

Choosing the Lyapunov function *V*_1_ as

$$V_{1}=\frac{1}{2}z_{1}^{2}+\frac{1}{2a_{1}}{\tilde{W}}_{1}^{T}{\tilde{W}}_{1}+\frac{1}{2r_{1}}{\tilde{D}}_{1}^{2}+\frac{1}{2}\beta_{1}^{2}$$ (18)

where ${\tilde{W}}_{1}=W_{1}^{*}-{\hat{W}}_{1}$ and ${\tilde{D}}_{1}=D_{1}-{\hat{D}}_{1}$, ${\hat{W}}_{1}$ and ${\hat{D}}_{1}$ represent the estimation of $W_{1}^{*}$ and *D*_1_, *a*_1_>0 and *r*_1_>0 are design parameters. Then, the time derivative of *V*_1_ can be expressed as

$${\dot{V}}_{1}=z_{1}{\dot{z}}_{1}-\frac{1}{a_{1}}{\tilde{W}}_{1}^{T}{\dot{\hat{W}}}_{1}-\frac{1}{r_{1}}{\tilde{D}}_{1}{\dot{\hat{D}}}_{1}+\beta_{1}{\dot{\beta }}_{1}$$ (19)

Design the virtual control law *α*_1_, parameter adaptive updating control laws ${\hat{W}}_{1}$ and ${\hat{D}}_{1}$ as

$$\alpha_{1}=\frac{1}{g_{1}}(-k_{1}z_{1}-{\hat{W}}_{1}^{T}S_{1}(Z_{1})-{\hat{D}}_{1}\tanh(\frac{z_{1}}{\eta_{1}})+{\dot{y}}_{d}-c_{1}\lambda_{1})$$ (20)

$${\dot{\hat{W}}}_{1}=a_{1}z_{1}S_{1}(Z_{1})-\sigma_{1}{\hat{W}}_{1}$$ (21)

$${\dot{\hat{D}}}_{1}=r_{1}z_{1}\tanh(\frac{z_{1}}{\eta_{1}})-\upsilon_{1}{\hat{D}}_{1}$$ (22)

where *k*_1_>0, *σ*_1_>0 and *υ*_1_>0 are design parameters.

Substituting (17), (20)-(22) into (19), and considering Lemma 2, we have

$${\dot{V}}_{1}\leq g_{1}z_{1}z_{2}-k_{1}z_{1}^{2}+\frac{\sigma_{1}}{a_{1}}{\tilde{W}}_{1}^{T}{\hat{W}}_{1}+\frac{\upsilon_{1}}{r_{1}}{\tilde{D}}_{1}{\hat{D}}_{1}+g_{1}z_{1}\beta_{1}+\beta_{1}{\dot{\beta }}_{1}+0.2875\eta_{1}D_{1}$$ (23)

Further, using Lemma 1 and considering (15) and (16), we have

$$g_{1}z_{1}\beta_{1}\leq {\left(g_{1}z_{1}\right)}^{2}+\frac{\beta_{1}^{2}}{4}$$ (24)

$$\beta_{1}{\dot{\beta }}_{1}=-\frac{\beta_{1}^{2}}{\tau_{1}}-\beta_{1}{\dot{\alpha }}_{1}\leq -\frac{\beta_{1}^{2}}{\tau_{1}}+\frac{\beta_{1}^{2}B_{1}^{2}(\cdot )}{2}+\frac{1}{2}$$ (25)

where $B_{1}(\cdot )=B_{1}(x_{1},{\hat{W}}_{1},{\hat{D}}_{1},y_{d},{\dot{y}}_{d},{\ddot{y}}_{d})$ is a non-negative continuous function.

Substituting (24) and (25) into (23), we get

$${\dot{V}}_{1}\leq g_{1}z_{1}z_{2}-(k_{1}-g_{1}^{2})z_{1}^{2}+\frac{\sigma_{1}}{a_{1}}{\tilde{W}}_{1}^{T}{\hat{W}}_{1}+\frac{\upsilon_{1}}{r_{1}}{\tilde{D}}_{1}{\hat{D}}_{1}-(\frac{1}{\tau_{1}}-\frac{1}{4})\beta_{1}^{2}+\frac{\beta_{1}^{2}B_{1}^{2}(\cdot )}{2}+0.2875\eta_{1}D_{1}+\frac{1}{2}$$ (26)

**Step** *i* (*i* = 2,⋯,*n*−1). According to (1), ${\dot{\lambda }}_{i}=-c_{i}\lambda_{i}+g_{i}\lambda_{i+1}$ (*i* = 2,⋯,*n*−1) and (10), the time derivative of *z*_*i*_ is described as

$${\dot{z}}_{i}=g_{i}z_{i+1}+g_{i}{\bar{\alpha }}_{i}+f_{i}+d_{i}-{\dot{\bar{\alpha }}}_{i-1}+c_{i}\lambda_{i}$$ (27)

Noting *f*_*i*_ is an unknown nonlinear function, and an RBFNN is introduced to approximate it, then we have

$$f_{i}={\left(W_{i}^{*}\right)}^{T}S_{i}(Z_{i})+\varepsilon_{i}(Z_{i})$$ (28)

where *ε*_*i*_(*Z*_*i*_) is approximation error and satisfies $|\varepsilon_{i}(Z_{i})|\leq {\bar{\varepsilon }}_{i}$ with ${\bar{\varepsilon }}_{i}>0$.

Substituting (28) into (27) yields

$${\dot{z}}_{i}=g_{i}z_{i+1}+g_{i}{\bar{\alpha }}_{i}+{\left(W_{i}^{*}\right)}^{T}S_{i}(Z_{i})-{\dot{\bar{\alpha }}}_{i-1}+c_{i}\lambda_{i}+\varepsilon_{i}(Z_{i})+d_{i}$$ (29)

Due to $|\varepsilon_{i}(Z_{i})|\leq {\bar{\varepsilon }}_{i}$ and |*d*_*i*_|≤*d*_*iM*_, then $|\varepsilon_{i}(Z_{i})+d_{i}|\leq {\bar{\varepsilon }}_{i}+d_{iM}$ and let $D_{i}={\bar{\varepsilon }}_{i}+d_{iM}$, where *D*_*i*_ is an unknown positive constant.

To avoid the problem of complexity explosion caused by repeated differentiation, a first-order low-pass filter 1/(*τ*_*i*_*s*+1) with time constant *τ*_*i*_ being introduced, then we have

$$\tau_{i}{\dot{\bar{\alpha }}}_{i}+{\bar{\alpha }}_{i}=\alpha_{i},\alpha_{i}(0)={\bar{\alpha }}_{i}(0)$$ (30)

where the virtual control law *α*_*i*_ as the input and the newly introduced state ${\bar{\alpha }}_{i}$ as the output.

Defining the filtering error *β*_*i*_ as

$$\beta_{i}={\bar{\alpha }}_{i}-\alpha_{i}$$ (31)

Combining (30) and (31), we have

$${\dot{\bar{\alpha }}}_{i}=\frac{\alpha_{i}-{\bar{\alpha }}_{i}}{\tau_{i}}=-\frac{\beta_{i}}{\tau_{i}}$$ (32)

Considering ${\bar{\varepsilon }}_{i}+d_{iM}=D_{i}$ and substituting (31) into (29) yields

$${\dot{z}}_{i}\leq g_{i}z_{i+1}+g_{i}\alpha_{i}+{\left(W_{i}^{*}\right)}^{T}S_{i}(Z_{i})+g_{i}\beta_{i}-{\dot{\bar{\alpha }}}_{i-1}+c_{i}\lambda_{i}+D_{i}$$ (33)

Choosing the Lyapunov function *V*_*i*_ as

$$V_{i}=V_{i-1}+\frac{1}{2}z_{i}^{2}+\frac{1}{2a_{i}}{\tilde{W}}_{i}^{T}{\tilde{W}}_{i}+\frac{1}{2r_{i}}{\tilde{D}}_{i}^{2}+\frac{1}{2}\beta_{i}^{2}$$ (34)

where ${\tilde{W}}_{i}=W_{i}^{*}-{\hat{W}}_{i}$ and ${\tilde{D}}_{i}=D_{i}-{\hat{D}}_{i}$, ${\hat{W}}_{i}$ and ${\hat{D}}_{i}$ are the estimation of $W_{i}^{*}$ and *D*_*i*_, *a*_*i*_>0 and *r*_*i*_>0 are design parameters. So the time derivative of *V*_*i*_ can be expressed as

$${\dot{V}}_{i}={\dot{V}}_{i-1}+z_{i}{\dot{z}}_{i}-\frac{1}{a_{i}}{\tilde{W}}_{i}^{T}{\dot{\hat{W}}}_{i}-\frac{1}{r_{i}}{\tilde{D}}_{i}{\dot{\hat{D}}}_{i}+\beta_{i}{\dot{\beta }}_{i}$$ (35)

Design the virtual control law *α*_*i*_, parameter adaptive updating control laws ${\hat{W}}_{i}$ and ${\hat{D}}_{i}$ as

$$\alpha_{i}=\frac{1}{g_{i}}(-k_{i}z_{i}-{\hat{W}}_{i}^{T}S_{i}(Z_{i})-{\hat{D}}_{i}\tanh(\frac{z_{i}}{\eta_{i}})+{\dot{\alpha }}_{i-1}-c_{i}\lambda_{i}-g_{i-1}z_{i-1})$$ (36)

$${\dot{\hat{W}}}_{i}=a_{i}z_{i}S_{i}(Z_{i})-\sigma_{i}{\hat{W}}_{i}$$ (37)

$${\dot{\hat{D}}}_{i}=r_{i}z_{i}\tanh(\frac{z_{i}}{\eta_{i}})-\upsilon_{i}{\hat{D}}_{i}$$ (38)

where *k*_*i*_>0, *σ*_*i*_>0 and *υ*_*i*_>0 are design parameters.

Considering Lemma 2, and substituting (33), (36)-(38) into (35), we have

$${\dot{V}}_{i}\leq {\dot{V}}_{i-1}+g_{i}z_{i}z_{i+1}-g_{i-1}z_{i-1}z_{i}-k_{i}z_{i}^{2}+\frac{\sigma_{i}}{a_{i}}{\tilde{W}}_{i}^{T}{\hat{W}}_{i}+\frac{\upsilon_{i}}{r_{i}}{\tilde{D}}_{i}{\hat{D}}_{i}+g_{i}z_{i}\beta_{i}+\beta_{i}{\dot{\beta }}_{i}+0.2875\eta_{i}D_{i}$$ (39)

Further, using Lemma 1 and considering (31) and (32), we have

$$g_{i}z_{i}\beta_{i}\leq {\left(g_{i}z_{i}\right)}^{2}+\frac{\beta_{i}^{2}}{4}$$ (40)

$$\beta_{i}{\dot{\beta }}_{i}\leq -\frac{\beta_{i}^{2}}{\tau_{i}}+\frac{\beta_{i}^{2}B_{i}^{2}(\cdot )}{2}+\frac{1}{2}$$ (41)

where $B_{i}(\cdot )=B_{i}(x_{1},\cdots x_{i},{\hat{W}}_{i},{\hat{D}}_{i},y_{d},{\dot{y}}_{d},\cdots ,y_{d}^{\left(i+1\right)})$ is a non-negative continuous function.

Considering the result of ${\dot{V}}_{i-1}$ in step *i*−1, we have

$$\begin{array}{l} {\dot{V}}_{i-1}\leq g_{i-1}z_{i-1}z_{i}-\sum_{m=1}^{i-1}(k_{m}-g_{m}^{2})z_{m}^{2}+\sum_{m=1}^{i-1}\frac{\sigma_{m}}{a_{m}}{\tilde{W}}_{m}^{T}{\hat{W}}_{m}+\sum_{m=1}^{i-1}\frac{\upsilon_{m}}{r_{m}}{\tilde{D}}_{m}{\hat{D}}_{m}-\sum_{m=1}^{i-1}(\frac{1}{\tau_{m}}-\frac{1}{4})\beta_{m}^{2}\\ \;+\sum_{m=1}^{i-1}\frac{\beta_{m}^{2}B_{m}^{2}(\cdot )}{2}+\sum_{m=1}^{i-1}0.2875\eta_{m}D_{m}+\frac{1}{2}(i-1) \end{array}$$ (42)

Substituting (40)-(42) into (39), we obtain

$$\begin{array}{l} {\dot{V}}_{i}\leq g_{i}z_{i}z_{i+1}-\sum_{m=1}^{i}(k_{m}-g_{m}^{2})z_{m}^{2}+\sum_{m=1}^{i}\frac{\sigma_{m}}{a_{m}}{\tilde{W}}_{m}^{T}{\hat{W}}_{m}+\sum_{m=1}^{i}\frac{\upsilon_{m}}{r_{m}}{\tilde{D}}_{m}{\hat{D}}_{m}-\sum_{m=1}^{i}(\frac{1}{\tau_{m}}-\frac{1}{4})\beta_{m}^{2}\\ \;+\sum_{m=1}^{i}\frac{\beta_{m}^{2}B_{m}^{2}(\cdot )}{2}+\sum_{m=1}^{i}0.2875\eta_{m}D_{m}+\frac{1}{2}i \end{array}$$ (43)

**Step** *n*. In this step, the actual control law will be given. According to (1), ${\dot{\lambda }}_{n}=-c_{n}\lambda_{n}+g_{n}\Delta u$, Δ*u* = *Q*(*u*(*t*))−*u*(*t*) and (10), the time derivative of *z*_*n*_ is given as

$${\dot{z}}_{n}=g_{n}u(t)+f_{n}+d_{n}-{\dot{\bar{\alpha }}}_{n-1}+c_{n}\lambda_{n}$$ (44)

Noting *f*_*n*_ is an unknown nonlinear function, and an RBFNN is introduced to approximate it, then we have

$$f_{n}={\left(W_{n}^{*}\right)}^{T}S_{n}(Z_{n})+\varepsilon_{n}(Z_{n})$$ (45)

where *ε*_*n*_(*Z*_*n*_) is the approximation error and satisfies $|\varepsilon_{n}(Z_{n})|\leq {\bar{\varepsilon }}_{n}$ with ${\bar{\varepsilon }}_{n}>0$.

Substituting (45) into (44) has

$${\dot{z}}_{n}=g_{n}u(t)+{\left(W_{n}^{*}\right)}^{T}S_{n}(Z_{n})-{\dot{\bar{\alpha }}}_{n-1}+c_{n}\lambda_{n}+\varepsilon_{n}(Z_{n})+d_{n}$$ (46)

Due to $|\varepsilon_{n}(Z_{n})|\leq {\bar{\varepsilon }}_{n}$ and |*d*_*n*_|≤*d*_*nM*_, then $|\varepsilon_{n}(Z_{n})+d_{n}|\leq {\bar{\varepsilon }}_{n}+d_{nM}$ and let $D_{n}={\bar{\varepsilon }}_{n}+d_{nM}$, where *D*_*n*_ is an unknown positive constant.

Choosing the Lyapunov *V*_*n*_ as

$$V_{n}=V_{n-1}+\frac{1}{2}z_{n}^{2}+\frac{1}{2a_{n}}{\tilde{W}}_{n}^{T}{\tilde{W}}_{n}+\frac{1}{2r_{n}}{\tilde{D}}_{n}^{2}$$ (47)

where ${\tilde{W}}_{n}=W_{n}^{*}-{\hat{W}}_{n}$ and ${\tilde{D}}_{n}=D_{n}-{\hat{D}}_{n}$, ${\hat{W}}_{n}$ and ${\hat{D}}_{n}$ represent the estimation of $W_{n}^{*}$ and *D*_*n*_, *a*_*n*_>0 and *r*_*n*_>0 are design parameters. So the time derivative of *V*_*n*_ is

$${\dot{V}}_{n}={\dot{V}}_{n-1}+z_{n}{\dot{z}}_{n}-\frac{1}{a_{n}}{\tilde{W}}_{n}^{T}{\dot{\hat{W}}}_{n}-\frac{1}{r_{n}}{\tilde{D}}_{n}{\dot{\hat{D}}}_{n}$$ (48)

Design the adaptive NN dynamic surface controller *u*(*t*), adaptive updating control laws ${\hat{W}}_{n}$ and ${\hat{D}}_{n}$ as

$$u(t)=\frac{1}{g_{n}}(-k_{n}z_{n}-{\hat{W}}_{n}^{T}S_{n}(Z_{n})-{\hat{D}}_{n}\tanh(\frac{z_{n}}{\eta_{n}})+{\dot{\alpha }}_{n-1}-c_{n}\lambda_{n}-g_{n-1}z_{n-1})$$ (49)

$${\dot{\hat{W}}}_{n}=a_{n}z_{n}S_{n}(Z_{n})-\sigma_{n}{\hat{W}}_{n}$$ (50)

$${\dot{\hat{D}}}_{n}=r_{n}z_{n}\tanh(\frac{z_{n}}{\eta_{n}})-\upsilon_{n}{\hat{D}}_{n}$$ (51)

where *k*_*n*_>0, *σ*_*n*_>0 and *υ*_*n*_>0 are design parameters.

Considering the result of ${\dot{V}}_{n-1}$ in step *n*−1, we have

$$\begin{array}{l} {\dot{V}}_{n-1}\leq g_{n-1}z_{n-1}z_{n}-\sum_{m=1}^{n-1}(k_{m}-g_{m}^{2})z_{m}^{2}+\sum_{m=1}^{n-1}\frac{\sigma_{m}}{a_{m}}{\tilde{W}}_{m}^{T}{\hat{W}}_{m}+\sum_{m=1}^{n-1}\frac{\upsilon_{m}}{r_{m}}{\tilde{D}}_{m}{\hat{D}}_{m}-\sum_{m=1}^{n-1}(\frac{1}{\tau_{m}}-\frac{1}{4})\beta_{m}^{2}\\ \;+\sum_{m=1}^{n-1}\frac{\beta_{m}^{2}B_{m}^{2}(\cdot )}{2}+\sum_{m=1}^{n-1}0.2875\eta_{m}D_{m}+\frac{1}{2}(n-1) \end{array}$$ (52)

Considering Lemma 2 and ${\bar{\varepsilon }}_{n}+d_{nM}=D_{n}$, and substituting (46), and (49)-(52) into (48), we have

$$\begin{array}{l} {\dot{V}}_{n}\leq -\sum_{m=1}^{n-1}(k_{m}-g_{m}^{2})z_{m}^{2}-k_{n}z_{n}^{2}+\sum_{m=1}^{n}\frac{\sigma_{m}}{a_{m}}{\tilde{W}}_{m}^{T}{\hat{W}}_{m}+\sum_{m=1}^{n}\frac{\upsilon_{m}}{r_{m}}{\tilde{D}}_{m}{\hat{D}}_{m}-\sum_{m=1}^{n-1}(\frac{1}{\tau_{m}}-\frac{1}{4})\beta_{m}^{2}\\ \;+\sum_{m=1}^{n-1}\frac{\beta_{m}^{2}B_{m}^{2}(\cdot )}{2}+\sum_{m=1}^{n}0.2875\eta_{m}D_{m}+\frac{1}{2}(n-1) \end{array}$$ (53)

### Stability analysis

According to the above analysis, the main results of this work can be summarized as follows.

#### Theorem 1

Under the Assumptions 1–3, considering the strict-feedback nonlinear system with input saturation nonlinearity (1), using the virtual control laws (20) and (36) with adaptive updating control laws (21), (22), (37) and (38), and the adaptive NN dynamic surface controller (49) with adaptive updating control laws (50) and (51), then it can be obtained that the output of the system output can track the reference trajectory and the tracking error can converge to a small neighborhood of zero, and all signals of the closed-loop system are bounded.

**Proof.** According to Lemma 1 and considering (53), we have

$$\frac{\sigma_{m}}{a_{m}}{\tilde{W}}_{m}^{T}{\hat{W}}_{m}\leq -\frac{\sigma_{m}}{2a_{m}}{\tilde{W}}_{m}^{T}{\tilde{W}}_{m}+\frac{\sigma_{m}}{2a_{m}}W_{m}^{T}W_{m}$$ (54)

$$\frac{\upsilon_{m}}{r_{m}}{\tilde{D}}_{m}{\hat{D}}_{m}\leq -\frac{\upsilon_{m}}{2r_{m}}{\tilde{D}}_{m}^{2}+\frac{\upsilon_{m}}{2r_{m}}D_{m}^{2}$$ (55)

Further, the following compact set is defined as

$$\Omega_{i}≔\{\begin{array}{l} z_{1},\cdots ,z_{i},{\hat{W}}_{1},\cdots ,{\hat{W}}_{i},{\hat{D}}_{1},\cdots ,{\hat{D}}_{i},\beta_{1},\cdots ,\beta_{i-1}:\\ \;\sum_{m=1}^{i}\left(z_{m}^{2}+{\tilde{W}}_{m}^{T}{\tilde{W}}_{m}+{\tilde{D}}_{m}^{2}\right)+\sum_{m=1}^{i-1}\beta_{m}^{2}\leq 2\Xi_{2},i=2,\cdots ,n \end{array}\}$$

According to Assumption 2, it can be inferred that Ω_1_ is a compact set. Therefore, we can obtain that all variables in the non-negative continuous functions *B*_*i*_(∙)(*i* = 1,⋯,*n*−1) are bounded within the compact set Ω_1_×Ω_i_. This also implies that *B*_*i*_(∙) are bounded within the compact set Ω_1_×Ω_*i*_, and further meaning that |*B*_*i*_(∙)| must exist a maximum value. Without loss of generality, let $\max|B_{i}(\cdot )|\leq {\bar{B}}_{iM}$.

Considering $\max|B_{i}(\cdot )|\leq {\bar{B}}_{iM}$ and substituting (54) and (55) into (53), we have

$$\begin{array}{l} {\dot{V}}_{n}\leq -\sum_{m=1}^{n-1}(k_{m}-g_{m}^{2})z_{m}^{2}-k_{n}z_{n}^{2}-\sum_{m=1}^{n}\frac{\sigma_{m}}{2a_{m}}{\tilde{W}}_{m}^{T}{\tilde{W}}_{m}-\sum_{m=1}^{n}\frac{\upsilon_{m}}{2r_{m}}{\tilde{D}}_{m}^{2}-\sum_{m=1}^{n-1}(\frac{1}{\tau_{m}}-\frac{1}{4}-\frac{{\bar{B}}_{mM}^{2}}{2})\beta_{m}^{2}\\ \;+\sum_{m=1}^{n-1}(\frac{B_{m}^{2}(\cdot )}{2}-\frac{{\bar{B}}_{mM}^{2}}{2})\beta_{m}^{2}+\sum_{m=1}^{n}\frac{\sigma_{m}}{2a_{m}}W_{m}^{T}W_{m}+\sum_{m=1}^{n}\frac{\upsilon_{m}}{2r_{m}}D_{m}^{2}+\sum_{m=1}^{n}0.2875\eta_{m}D_{m}+\frac{1}{2}(n-1) \end{array}$$ (56)

Noting

$$(\frac{B_{m}^{2}(\cdot )}{2}-\frac{{\bar{B}}_{mM}^{2}}{2})\beta_{m}^{2}\leq 0$$

Additionally, let

$$\vartheta =\min\{\begin{array}{l} 2(k_{1}-g_{1}^{2}),\cdots ,2(k_{n-1}-g_{n-1}^{2}),2k_{n},\sigma_{1},\cdots ,\sigma_{n},\upsilon_{1},\cdots ,\upsilon_{n},\\ \;\frac{2}{\tau_{1}}-\frac{1}{2}-B_{1M}^{2},\cdots ,\frac{2}{\tau_{n-1}}-\frac{1}{2}-B_{n-1,M}^{2} \end{array}\}$$

$$\omega =\sum_{m=1}^{n}\frac{\sigma_{m}}{2a_{m}}W_{m}^{T}W_{m}+\sum_{m=1}^{n}\frac{\upsilon_{m}}{2r_{m}}D_{m}^{2}+\sum_{m=1}^{n}0.2875\eta_{m}D_{m}+\frac{1}{2}(n-1)$$

Hence, the Eq (56) can be simplified as

$${\dot{V}}_{n}\leq -\vartheta V_{n}+\omega$$ (57)

Multiplying both sides of (57) by exp(*ϑt*) and integrating on (0,*t*] yields

$$V_{n}(t)\leq (V_{n}(0)-\frac{\omega }{\vartheta })\exp(-\vartheta t)+\frac{\omega }{\vartheta }\leq V_{n}(0)\exp(-\vartheta t)+\frac{\omega }{\vartheta }$$ (58)

As can be seen from (58), variables *z*_*i*_, ${\hat{W}}_{i}$, ${\hat{D}}_{i}$ and *β*_*j*_(*i* = 1,⋯.*n*, *j* = 1,⋯.*n*−1) are all bounded. Furthermore, it can be concluded that *α*_*j*_(*j* = 1,⋯.*n*−1) and *u*(*t*) are also bounded.

Combining (18), (34), and (47), it can be seen that

$$\frac{1}{2}z_{1}^{2}\leq V_{n}(0)\exp(-\vartheta t)+\frac{\omega }{\vartheta }$$ (59)

Furthermore, there are

$$\underset{t\to \infty }{\lim}|z_{1}|\leq \sqrt{\frac{2\omega }{\vartheta }}$$ (60)

Owing to |*x*_1_−*y*_*d*_|=|*z*_1_+*λ*_1_|≤|*z*_1_|+|*λ*_1_|, to prove that |*x*_1_−*y*_*d*_| is bounded, we only need to prove that |*λ*_1_| is bounded. The proof of the boundedness of |*λ*_1_| is shown below.

Taking the Lyapunov function $V_{\lambda }=\sum_{i=1}^{n}\lambda_{i}^{2}/2$. Considering (9), then the time derivative of *V*_*λ*_ is

$$\begin{array}{l} {\dot{V}}_{\lambda }=\sum_{i=1}^{n-1}\lambda_{i}(g_{i}\lambda_{i+1}-c_{i}\lambda_{i})-c_{n}\lambda_{n}^{2}+g_{n}\Delta u\lambda_{n}\\ \;\leq -\frac{1}{2}(2c_{1}-|g_{1}|)\lambda_{1}^{2}-\frac{1}{2}\sum_{i=2}^{n-1}(2c_{i}-|g_{i}|-|g_{i-1}|)\lambda_{i}^{2}-\frac{1}{2}(2c_{n}-|g_{n-1}|-\frac{1}{2})\lambda_{n}^{2}+{|g_{n}\Delta u|}^{2} \end{array}$$ (61)

According to Assumption 3, we can see that 0<*g*_*i*_≤*g*_0_(*i* = 1,⋯.*n*). Meanwhile, owing to *u*(*t*) being bounded and *Q*(*u*(*t*)) being the output of saturation fault, so it can be further obtained that Δ*u* is bounded, and thus |*g*_*n*_Δ*u*| is bounded.

Taking

$${\bar{\mu }}_{1}=2c_{1}-g_{0}$$

$${\bar{\mu }}_{i}=2c_{i}-2g_{0},i=2,\cdots ,n-1$$

$${\bar{\mu }}_{n}=2c_{n}-g_{0}-\frac{1}{2}$$

$$\mu =\min\{{\bar{\mu }}_{1},\cdots ,{\bar{\mu }}_{n}\}$$

Hence, (61) can be simplified as

$${\dot{V}}_{\lambda }\leq -\mu V_{\lambda }+A_{0}$$ (62)

where |*g*_*n*_Δ*u*|^2^≤*A*_0_.

Multiplying both sides of (62) by exp(*μt*) and integrating on (0,*t*] gets

$$V_{\lambda }(t)\leq V_{\lambda }(0)\exp(-\mu t)+\frac{A_{0}}{\mu }$$ (63)

Furthermore, we have

$$\underset{t\to \infty }{\lim}|\lambda_{1}|\leq \sqrt{\frac{2A_{0}}{\mu }}$$ (64)

It can be inferred from (64) that |*λ*_1_| is bounded. Taking into account (60) and (64), there is

$$\underset{t\to \infty }{\lim}|x_{1}-y_{d}|\leq \sqrt{\frac{2\omega }{\vartheta }}+\sqrt{\frac{2A_{0}}{\mu }}$$ (65)

It is not difficult to observe from (65) that by selecting appropriate parameters, the system tracking error |*x*_1_−*y*_*d*_| can be ensured to converge to a small neighborhood of zero. This completes the proof.

#### Remark 1

Observing (65), to adjust the size of the system tracking error, we can decrease the values of *ω* and *A*_0_, and on the other hand, we can also increase the values of *ϑ* and *μ*. By selecting these parameters appropriately, the tracking error can be ensured to be arbitrarily small.

#### Remark

For the parameters *ω*, *ϑ* and *μ*, on the one hand, decreasing *ω* can be achieved by increasing *a*_*m*_ and *r*_*m*_ or by decreasing *σ*_*m*_ and *υ*_*m*_, and increasing *ϑ* can be achieved by increasing *k*_*m*_, *σ*_*m*_ and *υ*_*m*_ or by decreasing *τ*_*j*_, and increasing *μ* can be achieved by increasing *c*_*m*_. On the other hand, however, the changes in *k*_*m*_, *c*_*m*_, *a*_*m*_, *r*_*m*_, *σ*_*m*_ and *υ*_*m*_(*m* = 1,⋯,*n*, *j* = 1,⋯,*n*−1) further affect the amplitude of virtual control laws, parameter update control laws, and actual controller signals. Therefore, a reasonable balance needs to be made between tracking performance and control signals.

## 4. Simulation analysis

In this section, two simulation cases are provided to demonstrate the effectiveness of the controller designed in this work.

**Case 1** (numerical case). Consider the following a type of strict-feedback nonlinear systems

$$\left\{ \begin{array}{l} {\dot{x}}_{1}=(0.5\sin(x_{1})+1)x_{2}+0.1\sin(x_{1})+0.5\sin(0.01t)\cos(0.02t)\\ {\dot{x}}_{2}=(0.5\cos(x_{1}x_{2})+1)Q(u(t))+x_{1}x_{2}^{2}+0.5\sin(0.01t)\cos(0.02t) \end{array}\right.$$ (66)

The auxiliary dynamic system is given as

$$\left\{ \begin{array}{l} {\dot{\lambda }}_{1}=-c_{1}\lambda_{1}+\lambda_{2}\\ {\dot{\lambda }}_{2}=-c_{2}\lambda_{2}+\Delta u\\ \Delta u=Q(u(t))-u(t) \end{array}\right.$$ (67)

where $f_{1}({\bar{x}}_{1})=0.1\sin(x_{1})$ and $f_{2}({\bar{x}}_{2})=x_{1}x_{2}^{2}$. For the input saturation nonlinearity (2), taking *U*_max_ = 6 and *U*_min_ = −6. The initial states of systems (66) and (67) are considered as *x*_1_(0) = 0.1, *x*_2_(0) = 0, and *λ*_1_(0) = *λ*_2_(0) = 0, respectively. The desired reference trajectory is given as *y*_*d*_ = 0.5(sin(*t*)+sin(0.5*t*), and simulation time is set as *t* = 40s.

The RBFNN for $f_{1}({\bar{x}}_{1})$, the number of nodes is set as 7, and its center *χ*_*i*_ is evenly spaced in the interval [−6,6]. The RBFNN for $f_{2}({\bar{x}}_{2})$, the number of nodes is set as 7, and its center *χ*_*i*_ is evenly spaced in the interval [−6,6]×[−6,6]. The width of both NNs is selected as *b*_*i*_ = 5.

Here, the virtual control law *α*_1_, the adaptive NN dynamic surface tracking controller *u*(*t*), and parameter adaptive updating control laws ${\hat{W}}_{i}$ and ${\hat{D}}_{i}$ (*i* = 1,2) are respectively given as

$$\alpha_{1}=\frac{1}{g_{1}}(-k_{1}z_{1}-{\hat{W}}_{1}^{T}S_{1}(Z_{1})-{\hat{D}}_{1}\tanh(\frac{z_{1}}{\eta_{1}})+{\dot{y}}_{d}-c_{1}\lambda_{1})$$

$$u(t)=\frac{1}{g_{2}}(-k_{2}z_{2}-{\hat{W}}_{2}^{T}S_{2}(Z_{2})-{\hat{D}}_{2}\tanh(\frac{z_{2}}{\eta_{2}})+{\dot{\bar{\alpha }}}_{1}-c_{2}\lambda_{2}-g_{1}z_{1})$$

$${\dot{\hat{W}}}_{i}=a_{i}z_{i}S_{i}(Z_{i})-\sigma_{i}{\hat{W}}_{i},i=1,2$$

$${\dot{\hat{D}}}_{i}=r_{i}z_{i}\tanh(\frac{z_{i}}{\eta_{i}})-\upsilon_{i}{\hat{D}}_{i},i=1,2$$

The simulation parameters are set as *τ*_1_ = 0.02, *η*_1_ = *η*_2_ = 0.05, *c*_1_ = *c*_2_ = 10, *k*_1_ = *k*_2_ = 9, *a*_1_ = *a*_2_ = 20, *σ*_1_ = *σ*_2_ = 15, *r*_1_ = *r*_2_ = 5, and *υ*_1_ = *υ*_2_ = 2. The initial states of parameter updating control laws are given as ${\hat{W}}_{1}(0)={\hat{W}}_{2}(0)={\left[0\right]}_{1\times 7}$ and ${\hat{D}}_{1}(0)={\hat{D}}_{2}(0)=0.1$.

**Fig 1:**
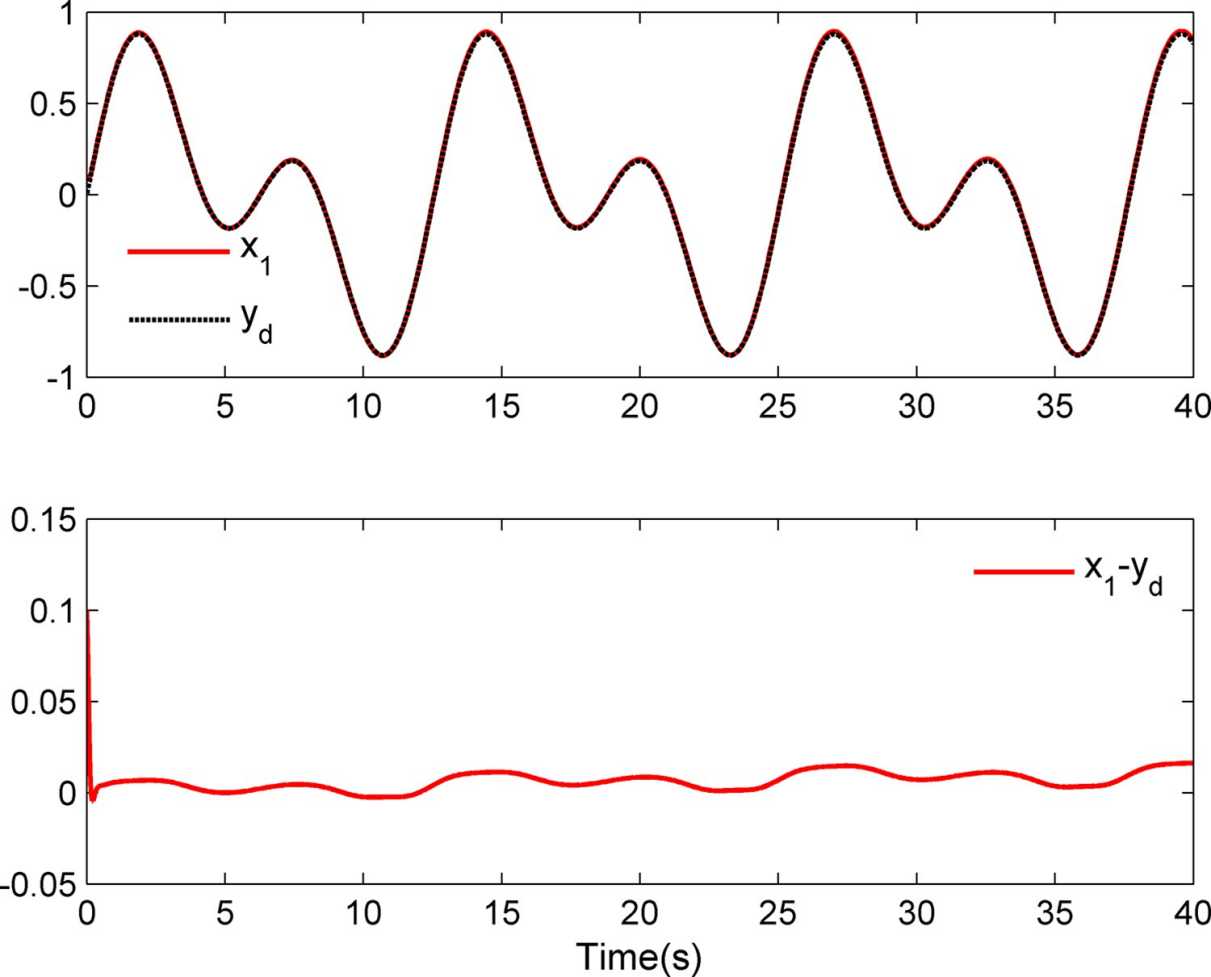
Tracking performance and tracking error *x*_1_–*y_d_*.

**Fig 2:**
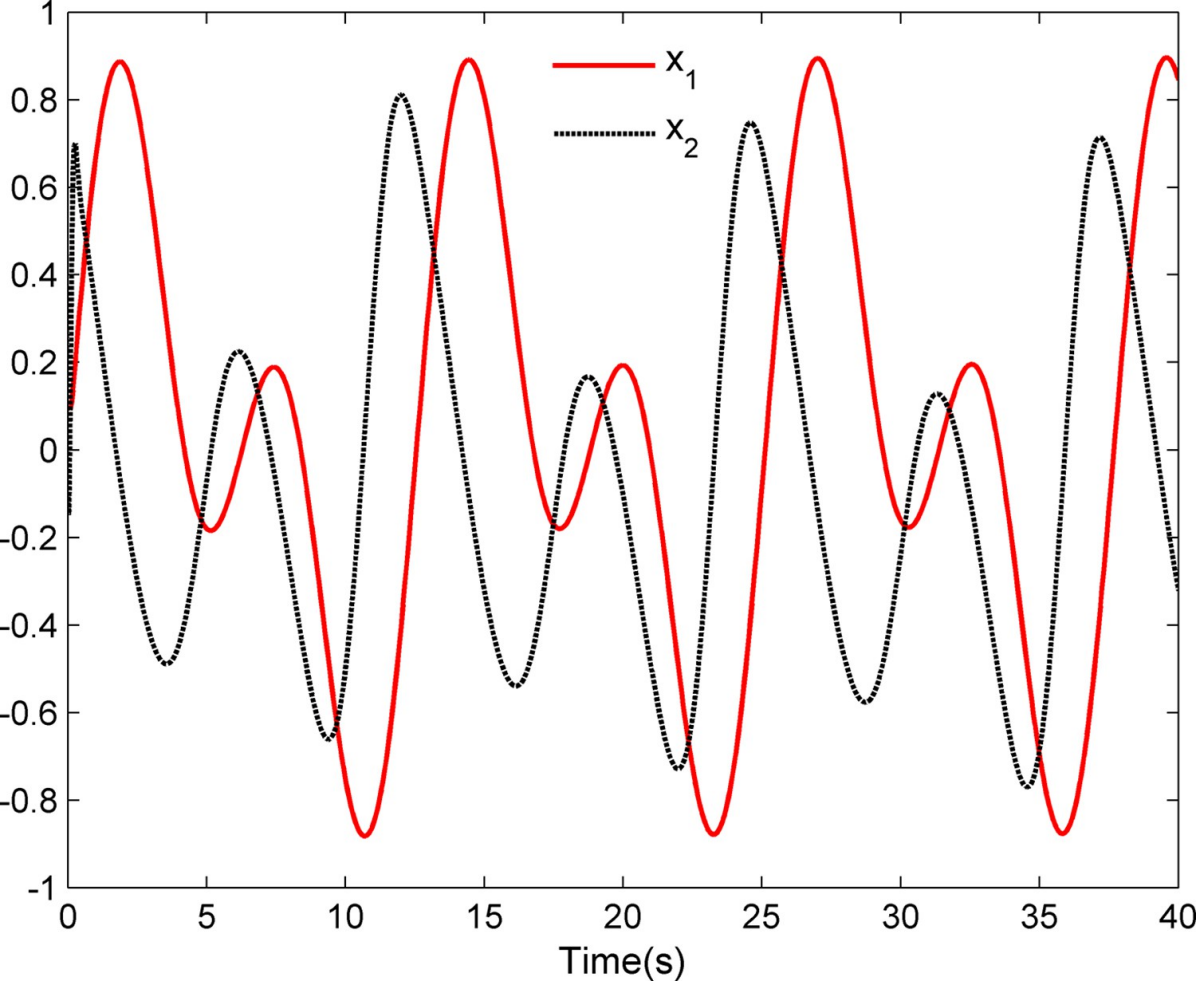
System states *x*_1_ and *x*_2_.

**Fig 3:**
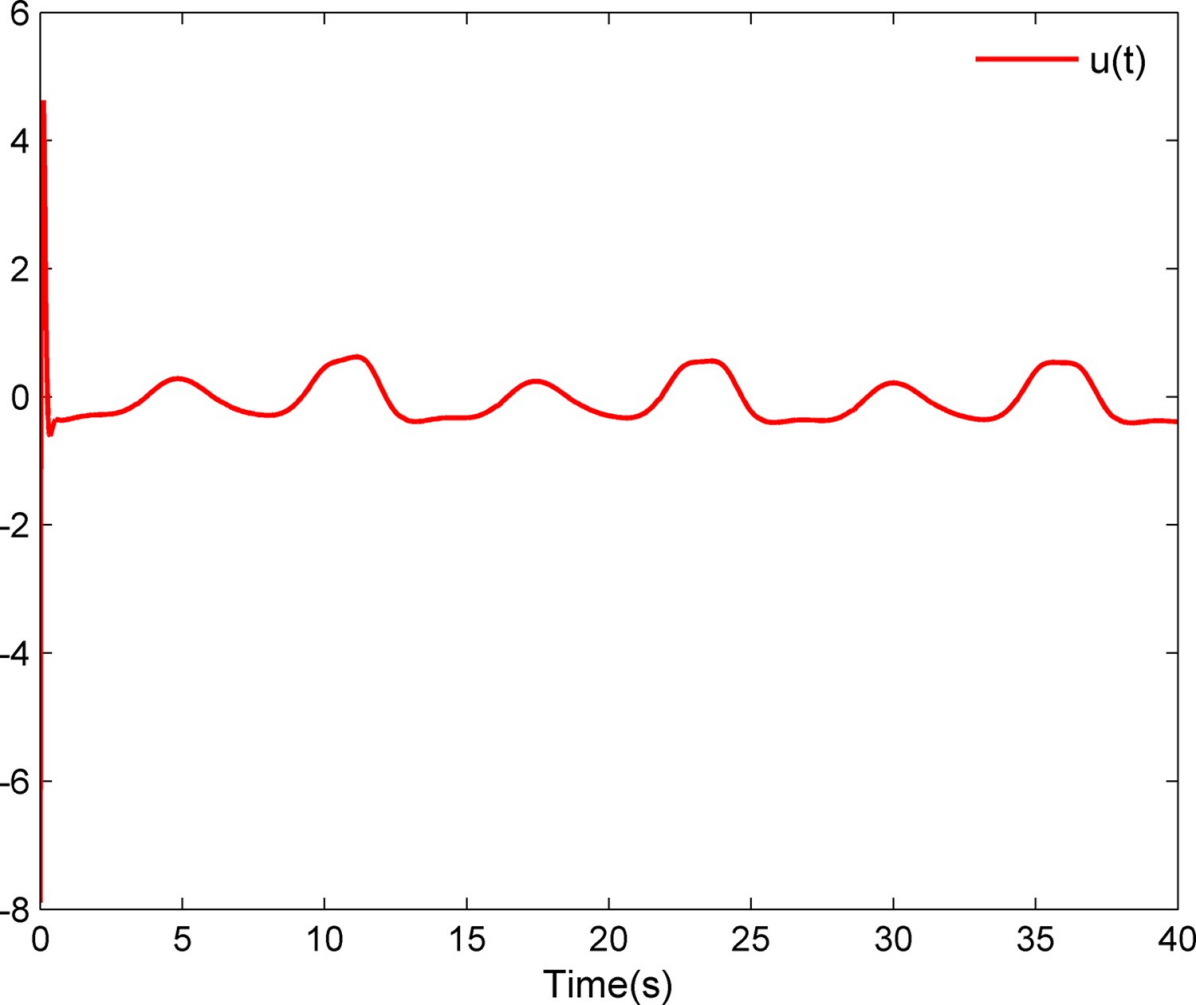
Controller *u*(*t*).

**Fig 4:**
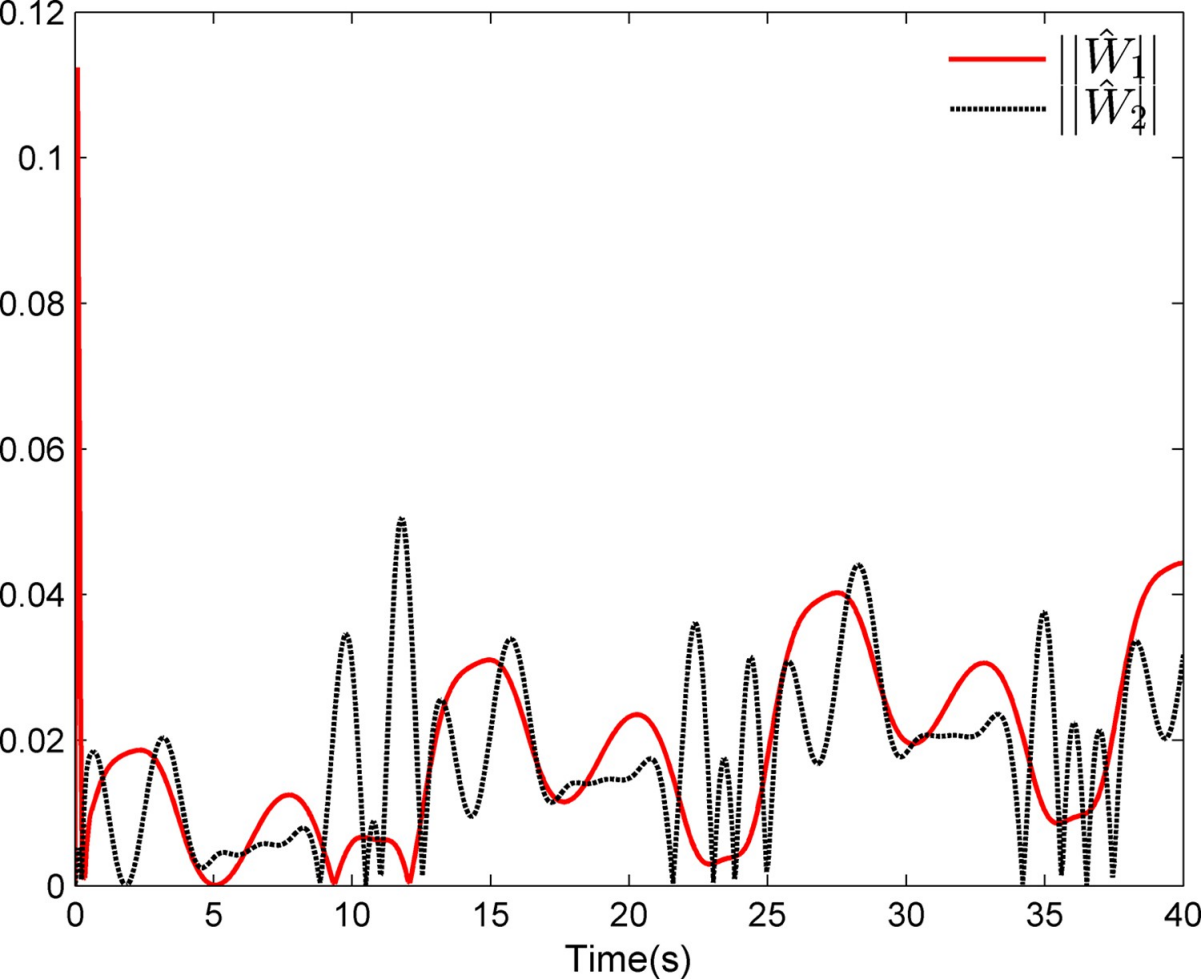
Adaptive updating control laws $\left\|{\hat{W}}_{1}\right\|$ and $\left\|{\hat{W}}_{2}\right\|$.

**Fig 5:**
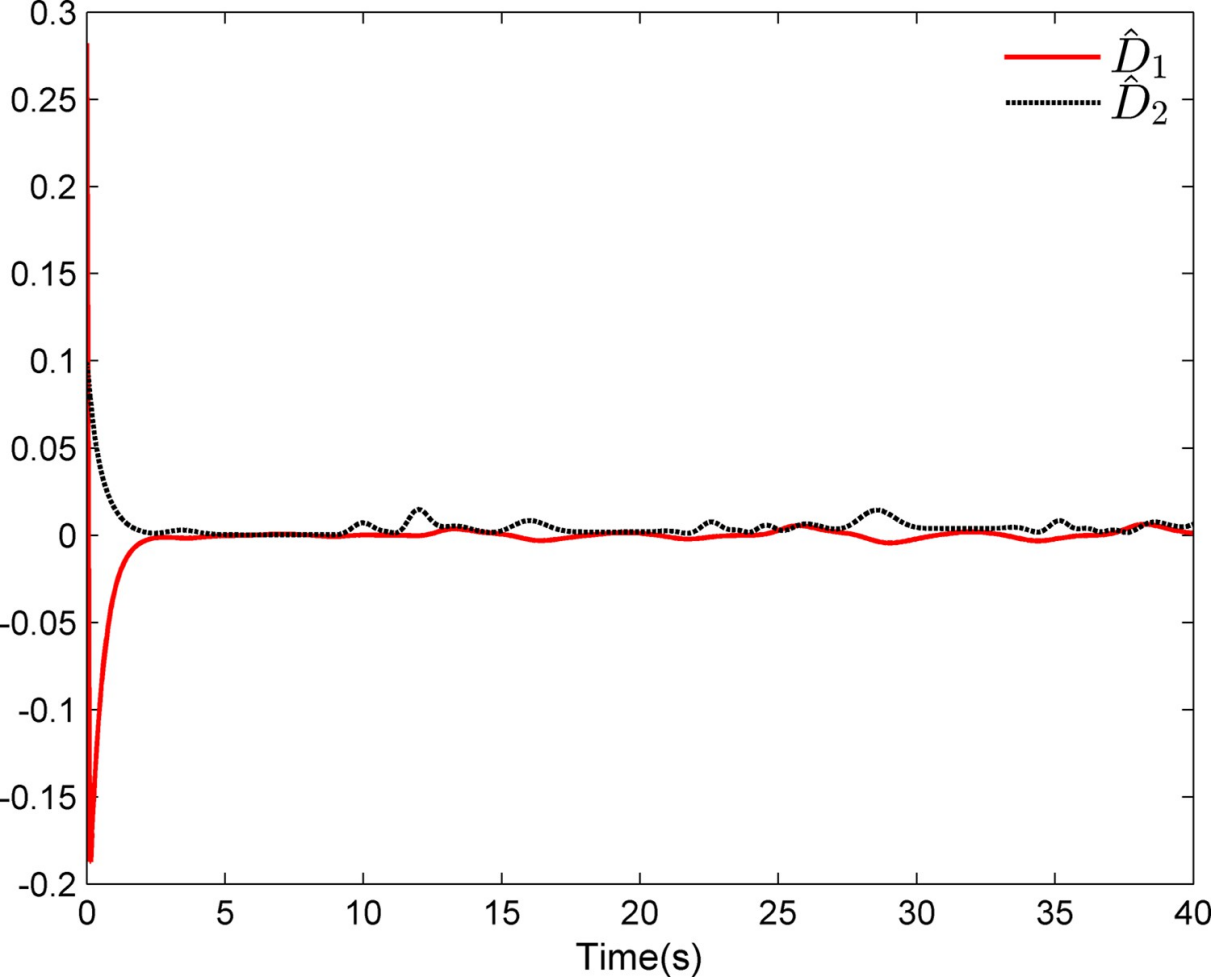
Adaptive updating control laws ${\hat{D}}_{1}$ and ${\hat{D}}_{2}$.

The simulation results appear in Figs 1–5. Fig 1 displays the results of tracking performance and tracking error. With the designed adaptive NN dynamic surface controller, the system output effectively tracks the reference trajectory, and the tracking error can converge to a small neighborhood of zero. Obviously, the controller designed in this work achieves favorable control effects, thereby confirming the correctness of the theoretical analysis. Additionally, the curves of system states *x*_*i*_, controller *u*(*t*), parameter adaptive updating control laws $\left\|{\hat{W}}_{i}\right\|$ and ${\hat{D}}_{i}$, *i* = 1,2, are respectively shown in Figs 2–5. From these figures, it is evident that all these signals remain bounded, which further reflects the effectiveness of the designed controller from another perspective.

Further, examining Figs 1–5, despite the given system being affected by input saturation nonlinearity, the designed controller and corresponding parameter adaptive updating control laws ensure that the tracking error of the system converges to a small neighborhood of zero. Furthermore, it is guaranteed that all variables of the closed-loop system are bounded.

**Case 2**. In order to further demonstrate the effectiveness of the controller proposed in this work, a type of actual system [37], namely a marine surface vehicle, is selected as the research plant of this case. The system model is described as:

$$\ddot{\phi }+\frac{K}{T}H(\dot{\phi })=\frac{K}{T}u(t)$$ (68)

where *ϕ* represents the yaw angle, *u*(*t*) represents the actuator angle, *K* and *T* represent hydrodynamic coefficients. $H(\dot{\phi })$ represents an unknown nonlinear function based on $\dot{\phi }$ which can be approximated by

$$H(\dot{\phi })=p_{1}\dot{\phi }+p_{2}{\dot{\phi }}^{3}+p_{3}{\dot{\phi }}^{5}$$ (69)

where *p*_1_, *p*_2_ and *p*_3_ are unknown parameters.

Assuming the system is affected by external disturbances and input saturation nonlinearity, and selecting *x*_1_ = *ϕ* and $x_{2}=\dot{\phi }$, then the system (68) can be converted into

$$\left\{ \begin{array}{l} {\dot{x}}_{1}=x_{2}+d_{1}(t)\\ {\dot{x}}_{2}=\frac{K}{T}Q(u(t))-\frac{K}{T}(p_{1}x_{2}+p_{2}x_{2}^{3}+p_{3}x_{2}^{5})+d_{2}(t) \end{array}\right.$$ (70)

where *d*_1_(*t*) and *d*_2_(*t*) represent external disturbances, *Q*(*u*(*t*) is the system input under the influence of input saturation nonlinearity.

Compared to the system (1), in this case, we have $f_{1}({\bar{x}}_{1})=0$ and $f_{2}({\bar{x}}_{2})=-K(p_{1}x_{2}+p_{2}x_{2}^{3}+p_{3}x_{2}^{5})/T$. Then, only $f_{2}({\bar{x}}_{2})$ is approximated by using RBFNN.

According to [37], the initial states are given as *x*_1_(0) = *x*_2_(0) = 0, *K* = 8, *T* = 2, *p*_1_ = *p*_2_ = *p*_3_ = 1. In addition, assuming the external disturbances of the system (68) are *d*_1_(*t*) = *d*_2_(*t*) = 0.5sin(0.1*t*) and *k*_1_ = *k*_2_ = 30. The selection of other parameters is the same as in Case 1.

**Fig 6:**
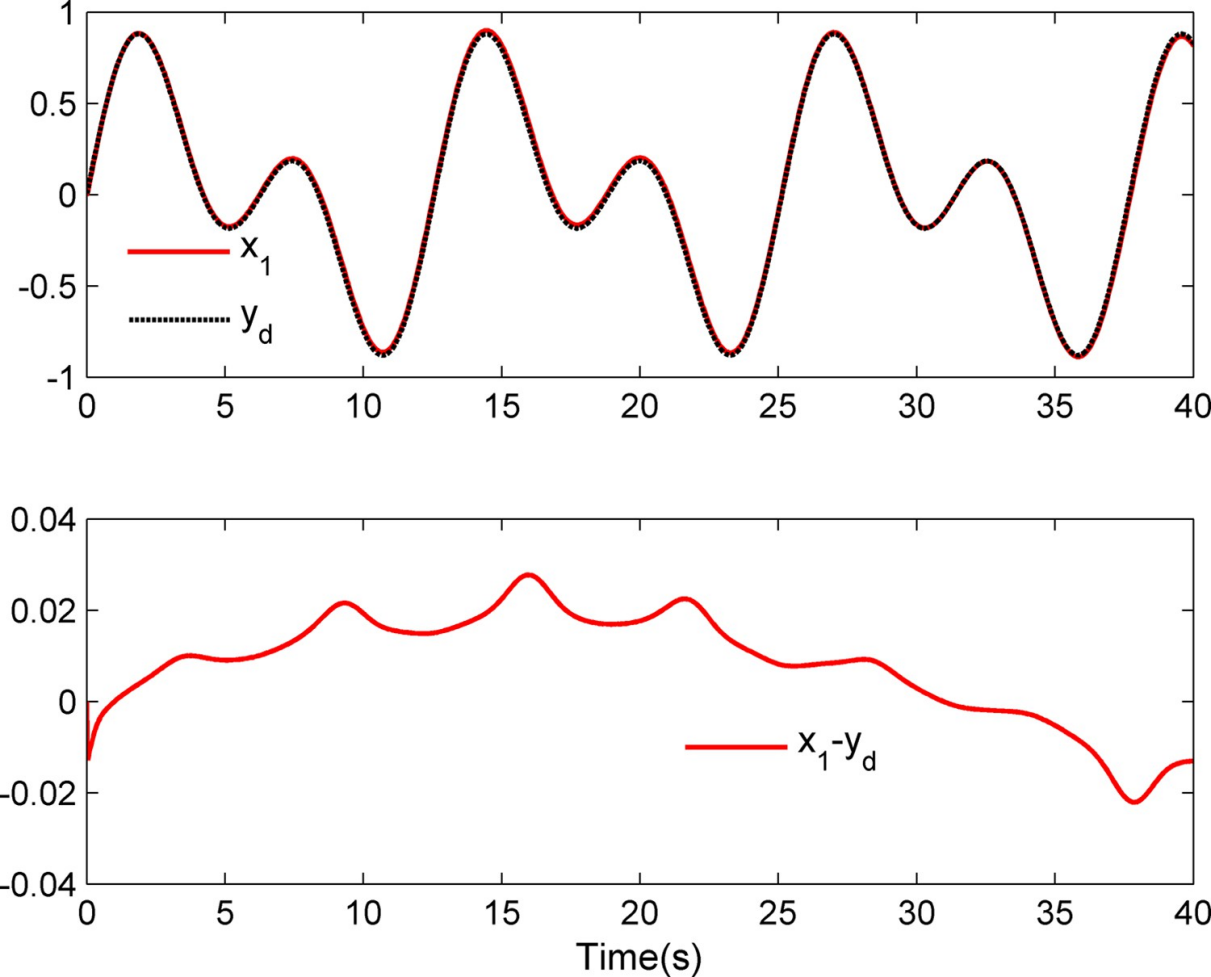
Tracking performance and tracking error *x*_1_–*y_d_*.

**Fig 7:**
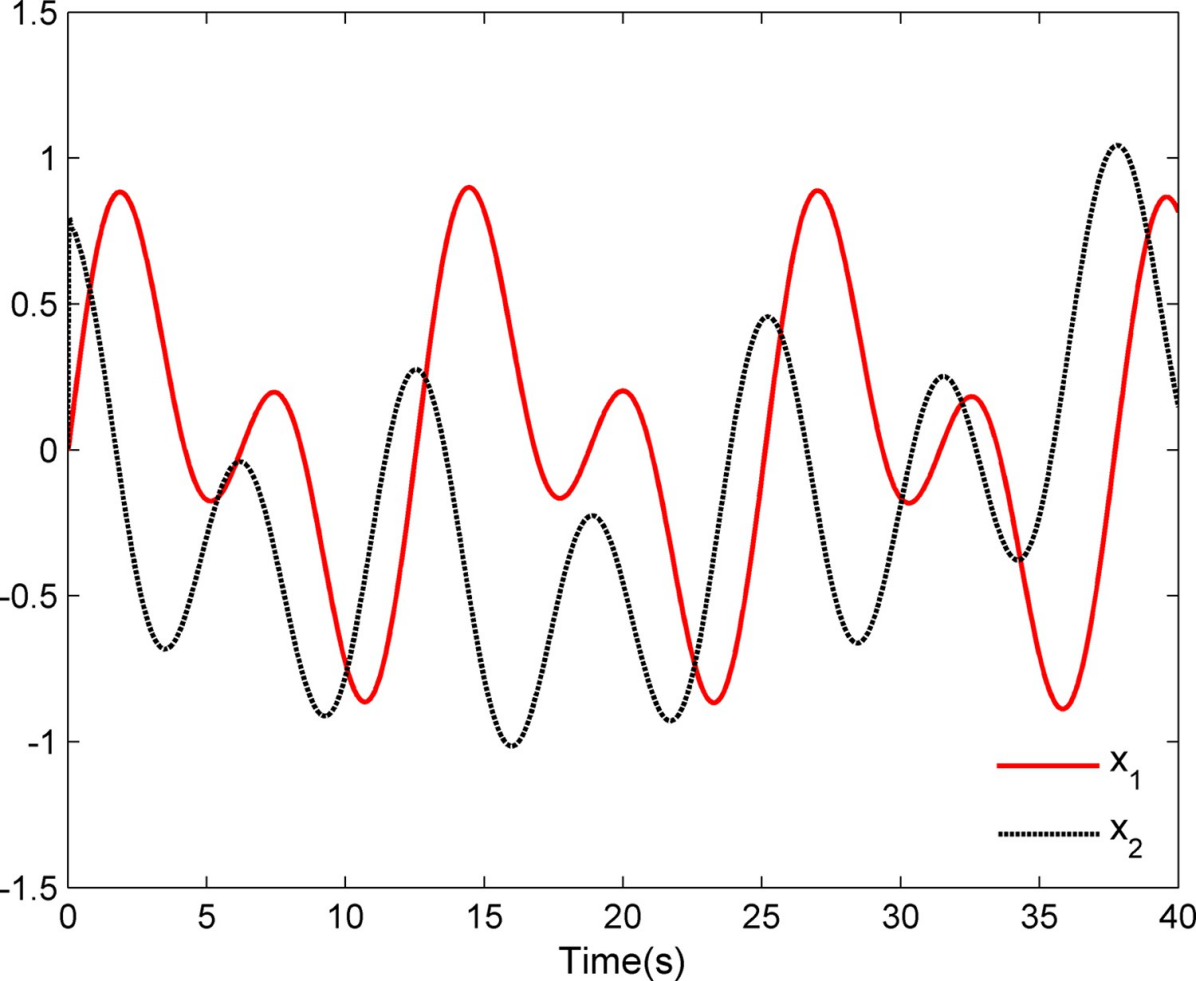
System states *x*_1_ and *x*_2_.

**Fig 8:**
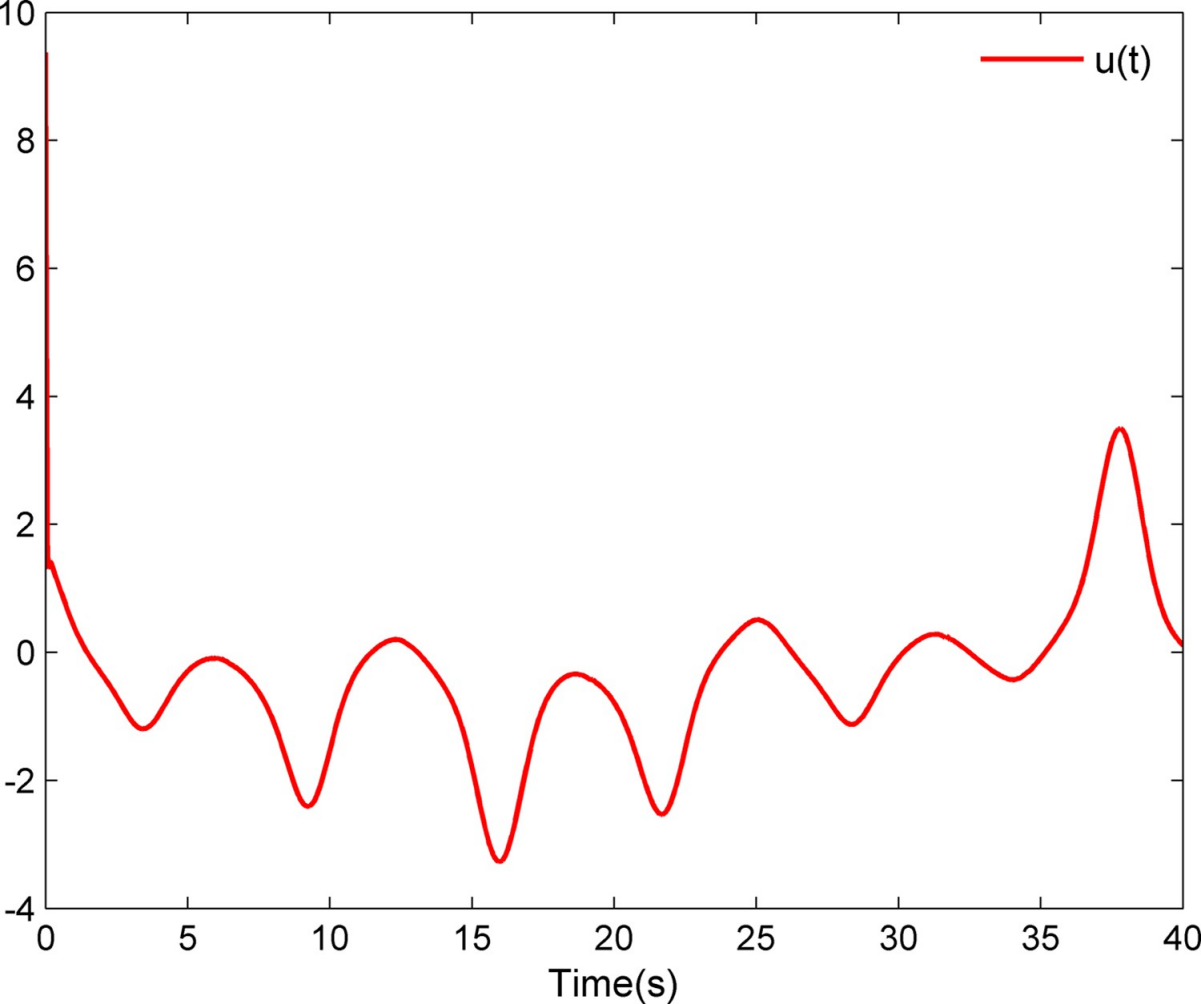
Controller *u*(*t*).

**Fig 9:**
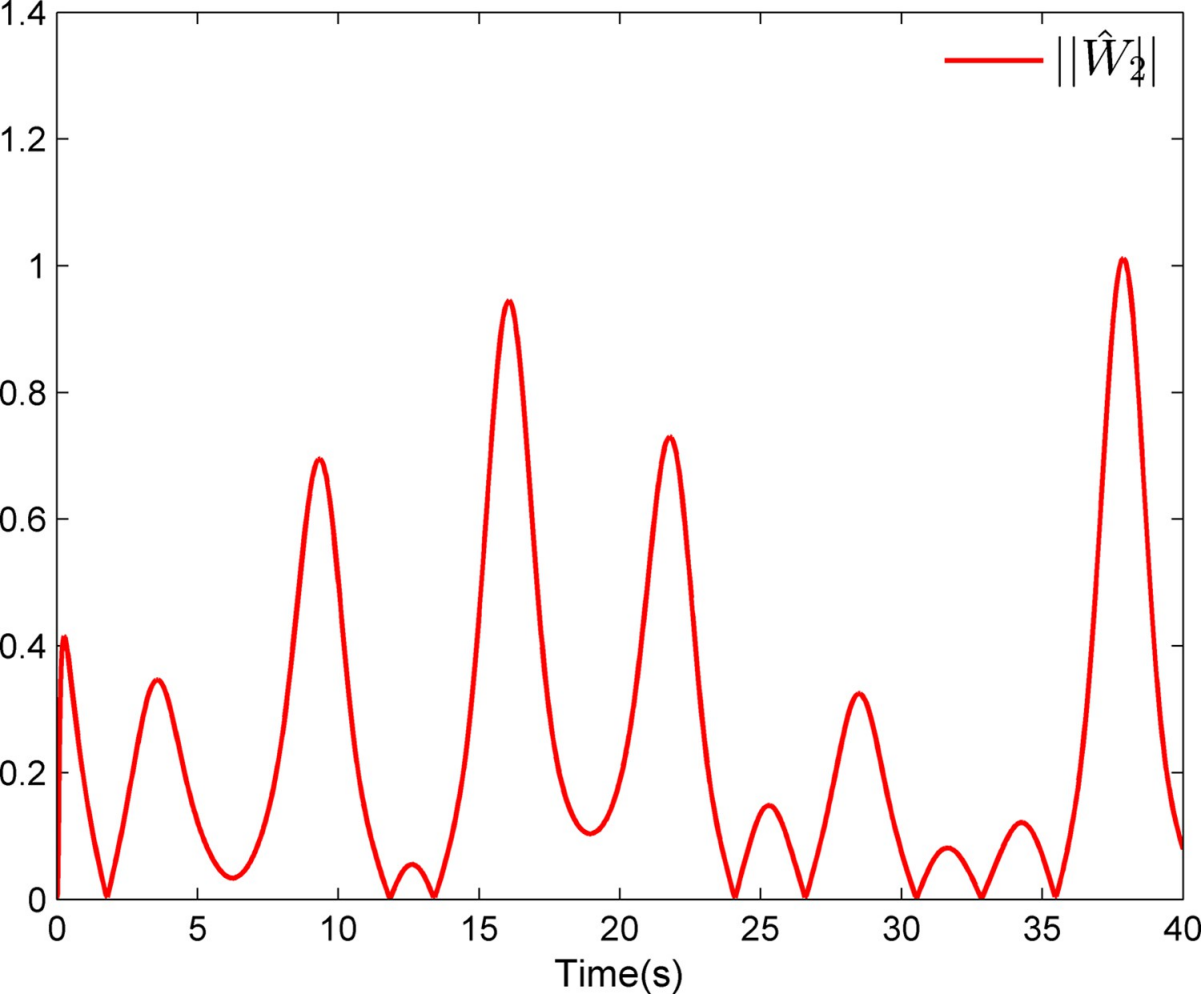
Adaptive updating control laws $\left\|{\hat{W}}_{2}\right\|$.

**Fig 10:**
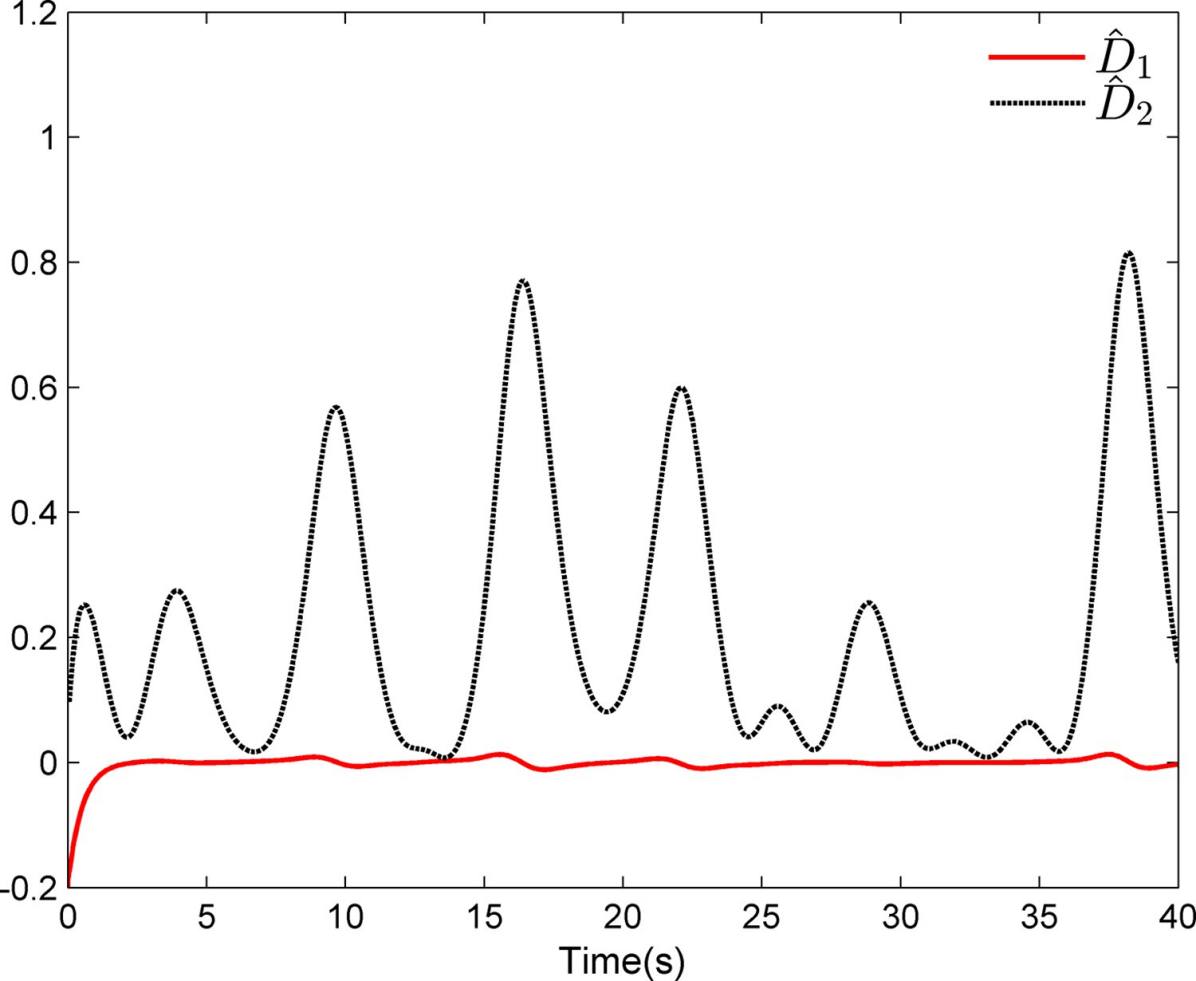
Adaptive updating control laws ${\hat{D}}_{1}$ and ${\hat{D}}_{2}$.

The simulation results display in Figs 6–10. Using the designed controller and parameter adaptive updating control laws, the curves of tracking performance and tracking error are depicted in Fig 6. It can be seen from the figure that the output of the marine surface vehicle system can effectively track the desired reference trajectory, and the tracking error can converge to a small neighborhood of zero. Moreover, Figs 6–10 show the system states, control input and parameter adaptive updating control laws, respectively. From these figures, one can observe that all variables remain bounded.

Furthermore, Figs 6–10 reveal that although the actual system experiences input saturation nonlinearity, the designed controller still achieves a strong control effect, which further proves the effectiveness of the designed controller.

## 5. Conclusion

This work studies the tracking control problem of a class of strict-feedback nonlinear systems with input saturation nonlinearity. An auxiliary control system is constructed to deal with the input saturation nonlinearity. In addition, DSC method and RBFNN approximate technique are applied for the controller design and the approximation of unknown nonlinear dynamics, respectively. Under the action of first-order low-pass filters, the problem of computational explosion caused by repeated differentiation of virtual control laws is effectively overcome. And then, an adaptive NN dynamic surface controller is designed for the tracking control problem. The simulation analysis verified the effectiveness of the designed controller. It can be ensured that the tracking error can converge to a small neighborhood of zero, and all signals of the closed-loop system remain bounded.

It should be pointed out that the control gains of the given system are assumed to be known, and only the influence of input saturation nonlinearity is considered. This greatly limits the application of the method proposed in this work. Therefore, in our follow work, we will consider the case of unknown gains or high-frequency gains, while also taking into account the effects of different input nonlinearities.
